# Early sepsis mortality prediction model based on interpretable machine learning approach: development and validation study

**DOI:** 10.1007/s11739-024-03732-2

**Published:** 2024-08-14

**Authors:** Yiping Wang, Zhihong Gao, Yang Zhang, Zhongqiu Lu, Fangyuan Sun

**Affiliations:** 1 Department of Emergency, The First Affiliated Hospital of WenZhou Medical University, Wenzhou, 325000 China; 2 Department of Computer Technology and Information Management, The First Affiliated Hospital of WenZhou Medical University, Wenzhou, 325000 China

**Keywords:** Machine learning, Artificial intelligence, XGBoost, Mortality prediction model, Sepsis, Septic shock

## Abstract

Sepsis triggers a harmful immune response due to infection, causing high mortality. Predicting sepsis outcomes early is vital. Despite machine learning’s (ML) use in medical research, local validation within the Medical Information Mart for Intensive Care IV (MIMIC-IV) database is lacking. We aimed to devise a prognostic model, leveraging MIMIC-IV data, to predict sepsis mortality and validate it in a Chinese teaching hospital. MIMIC-IV provided patient data, split into training and internal validation sets. Four ML models logistic regression (LR), support vector machine (SVM), deep neural networks (DNN), and extreme gradient boosting (XGBoost) were employed. Shapley additive interpretation offered early and interpretable mortality predictions. Area under the ROC curve (AUROC) gaged predictive performance. Results were cross verified in a Chinese teaching hospital. The study included 27,134 sepsis patients from MIMIC-IV and 487 from China. After comparing, 52 clinical indicators were selected for ML model development. All models exhibited excellent discriminative ability. XGBoost surpassed others, with AUROC of 0.873 internally and 0.844 externally. XGBoost outperformed other ML models (LR: 0.829; SVM: 0.830; DNN: 0.837) and clinical scores (Simplified Acute Physiology Score II: 0.728; Sequential Organ Failure Assessment: 0.728; Oxford Acute Severity of Illness Score: 0.738; Glasgow Coma Scale: 0.691). XGBoost’s hospital mortality prediction achieved AUROC 0.873, sensitivity 0.818, accuracy 0.777, specificity 0.768, and F1 score 0.551. We crafted an interpretable model for sepsis death risk prediction. ML algorithms surpassed traditional scores for sepsis mortality forecast. Validation in a Chinese teaching hospital echoed these findings.

## Introduction

Sepsis, a disordered immune reaction triggered by the body’s reaction to infection, has the potential to result in significant mortality rates [1]. Every year, millions of individuals worldwide are impacted by sepsis and septic shock, which pose significant challenges to healthcare [2]. The occurrence and death rates worldwide are increasing [3]. Policymakers consider the high expenses of intensive care unit (ICU) care and the frequent readmissions of sepsis survivors as a significant concern [1]. Hence, it is crucial to forecast the outcomes of sepsis patients promptly and precisely [4].

At present, various clinical scores such as the Sequential Organ Failure Assessment (SOFA), quick Sequential (Sepsis-related) Organ Failure Assessment (qSOFA) [5], Acute Physiology and Chronic Health Assessment Scoring System II (APACHE II), Oxford Acute Severity of Illness Score (OASIS) and Simplified Acute Physiology Score II (SAPS II) aid physicians in assessing the seriousness of sepsis and forecasting the likelihood of unfavorable incidents. Generally, the score rises as the patient’s likelihood of dying increases. Typically, these traditional measures of seriousness rely on a complex statistical model involving multiple variables and were designed for patients who are critically unwell in general, rather than specifically for sepsis. Simultaneously, these measures lack the accuracy needed for individual assessment, exhibiting considerable inaccuracies when applied to patient data that deviates from the mean [6]. It is practically impossible for humans to organize and interpret the vast amount of data collected from a patient in an ICU within the necessary timeframe, particularly when it comes to predicting the patient’s prognosis. Due to the inadequate effectiveness of current severity scores measures, several novel models have been created to forecast the likelihood of mortality among septic patients admitted to the ICU.

Over the past few years, there has been a gradual integration of machine learning (ML) into medical research [7]. ML has the ability to acquire knowledge from extensive medical data, investigate relationships within a dataset [8], and construct a relevant medical model capable of efficiently and precisely forecasting novel data [9].

The utilization of the Medical Information Mart for Intensive Care IV (MIMIC-IV) dataset in this research offers a chance to create a novel and enhanced model for predicting mortality, surpassing the previous studies that relied on the Mart for Intensive Care III (MIMIC-III) dataset. Our objective was to construct a risk forecasting algorithm utilizing the MIMIC-IV dataset to detect sepsis patients with a heightened likelihood of mortality upon admission to the hospital [10] and validate.

## Methods

In accordance with the Sepsis-3 definition, this research examined sepsis patients from the MIMIC-IV database, documenting vital signs, laboratory examinations, intervention therapies, and additional information [11]. In addition, we employed the Shapley additive explanation (SHAP) technique to elucidate the forecasting model [12]. This not only enables accurate prognosis prediction for patients but also offers a plausible justification for the prediction [11]. We have externally validated our model using a Chinese teaching hospital database, obtaining consistent outcomes.

### Data source

The research was carried out utilizing the MIMIC-IV dataset (version 1.0). MIMIC-IV comprises of 26 tables in a relational database. The MIMIC-IV dataset offers a possible benefit due to its increased magnitude and more up-to-date information in comparison to MIMIC-III [13, 14]. The Beth Israel Deaconess Medical Center has admitted more than 70,000 ICU patients between the years 2008 and 2019, and this database holds their data [15]. Authorized users were granted approval by the Massachusetts Institute of Technology (located in Cambridge, MA, USA) and the Institutional Review Board of BIDMC (based in Boston, MA, USA) to utilize the MIMIC-IV database [16]. Upon the successful fulfillment of the web-based training course offered by the National Institutes of Health (NIH) and the completion of the Protecting Human Research Participants examination [17] (Certification Number no.37920768), Yiping Wang was granted permission to retrieve data from the database. After receiving approval from the Hospital Ethics Committee (Issuing Number KY2022-R138) [18], external validation took place at the First Affiliated Hospital of Wenzhou Medical University (Wenzhou, Zhejiang, China).

### Patients and definitions

The screening process in this study employed the sepsis criteria established in 2016, known as Sepsis-3, to identify patients with sepsis. We initially chose patients who satisfied either of the following two conditions within a time frame ranging from 24 h prior to ICU admission to 24 h after: 1. By meticulously documenting infection-related diagnoses using the International Classification of Diseases, ninth revision, Clinical Modification (ICD-9-CM) codes, as provided by Angus et al. [19]; 2.Meeting the criteria for a suspected infection as outlined by Seymour CW et al. [20]. We then recognized subsequent organ malfunction as a sudden alteration in SOFA score of at least 2 points during the initial 24 h of being in the ICU. We only included the initial ICU admission for patients who had been admitted to the ICU two or more times during a single hospital stay [21]. Exclusion occurred for patients with a missing rate of predictor variable exceeding 25% in their records. The following criteria were used to exclude participants: (1) readmissions; (2) pregnant individuals; (3) ICU stays less than 24 h or longer than 100 days; (4) individuals under the age of 18 and (5) those with no access or incomplete data records. The external validation data were gathered from January 01, 2021, to June 30, 2022, following the identical criteria for inclusion and exclusion.

### Data collection and variable extraction

With reference to clinical expertise, published literature, and data records in the MIMIC-IV database [11], we gathered the subsequent seven categories of information. (1) Demographic details of the patient, such as gender, age, body mass index (BMI) and more; (2) The following should be assessed Within 24 h of being admitted to the ICU: essential signs including heart rate, average arterial pressure, respiratory rate, and Pao2/fio2 Ratio (P/F); (3) laboratory test outcomes within 24 h after admission to the ICU, such as creatinine, hemoglobin, albumin, lactate, and more; (4) treatment status within 24 h after entering the ICU, such as whether mechanical ventilation was performed, renal replacement treatment, etc.; (5) basic illnesses like hypertension, diabetes, chronic obstructive pulmonary disease (COPD), coronary kidney disease (CKD) and others; (6) outcome: ICU in-hospital mortality and (7) A variety of clinical scores within 24 h after ICU admission, including SOFA, SAPS II, and GCS [22]. For variables with multiple measurements in a day, we will in addition record the trend of each variable over time. We set different window lengths for each variable, take the average value in each window, and subtract the value of each window before the last window from the value of the last window, and finally divide it by the total number of windows to obtain the mean value of the variable’s trend over the day. To address anomalies in the data, we completely eliminate the apparent outliers. Multivariable imputations were employed to handle missing data. The estimators of the tree are set to 50 with PMM (candidate = 5) strategy, when comes to each field we assume the following Gaussian distribution and Poisson distribution. Additional file 3 included variables summary.

### Model establishment and evaluation

This research employed a combination of four ML models, namely logistic regression (LR), support vector machine (SVM), deep neural networks (DNN) and extreme gradient boosting (XGB). Considering the class imbalance, we adopted the way of Youden’s J statistic [23] to locate the optimal threshold of all the models. Additional file 1 included a concise overview of these fundamental ML models. We used the XGBoost model (Version 1.6.1). We utilized SHAP values to elucidate our initial predictive model and identify the risk factors associated with mortality in septic patients. The SHAP method offers a consistent and locally precise attribute value for each feature, making it a comprehensive approach to interpret the outcomes of any ML model. After computing the SHAP values for every feature in each sample, we identified the top 20 variables by considering the average SHAP values [12]. SHAP values are used to explain the output of ML models. They represent the contribution of each feature to the prediction made by the model. In other words, SHAP values help us understand how each input variable influences the model’s decision. Finally, a validation from an external source was conducted to confirm if comparable outcomes could be witnessed in a Chinese medical facility. Python version 3.9 was utilized for all computations and assessments, encompassing evaluation metrics like AUROC, specificity, sensitivity, and accuracy.

The training process involved the following steps: first, data from the MIMIC-IV database was utilized, and 70% of the cases were randomly selected as the training set, while the remaining 30% were assigned to the internal validation set [24]. Second, these trained models were used to predict the probabilities for the original samples in the internal validation set. Finally, the trained model was capable of predicting a new sample with new variables. Subsequently, the trained ML model was employed to forecast our hospital data, considering our local database as the external validation set.

### Statistical analysis

Measurement data that follows a normal distribution are typically represented as X ± S and are compared between groups using a two-independent-sample *t* test [25]. Data that are not normally distributed is represented as M (P25, P75) and is compared using the Mann–Whitney *U* test. The *χ*^2^ test was used to compare the groups by expressing the enumeration data in terms of rate and percentage [26]. The threshold for statistical significance was established at a *P*-value of less than 0.05. The SPSS software (version 26.0, Chicago, USA) was utilized for conducting statistical analyses [27].

## Results

### Baseline characteristics of study samples

**Fig. 1:**
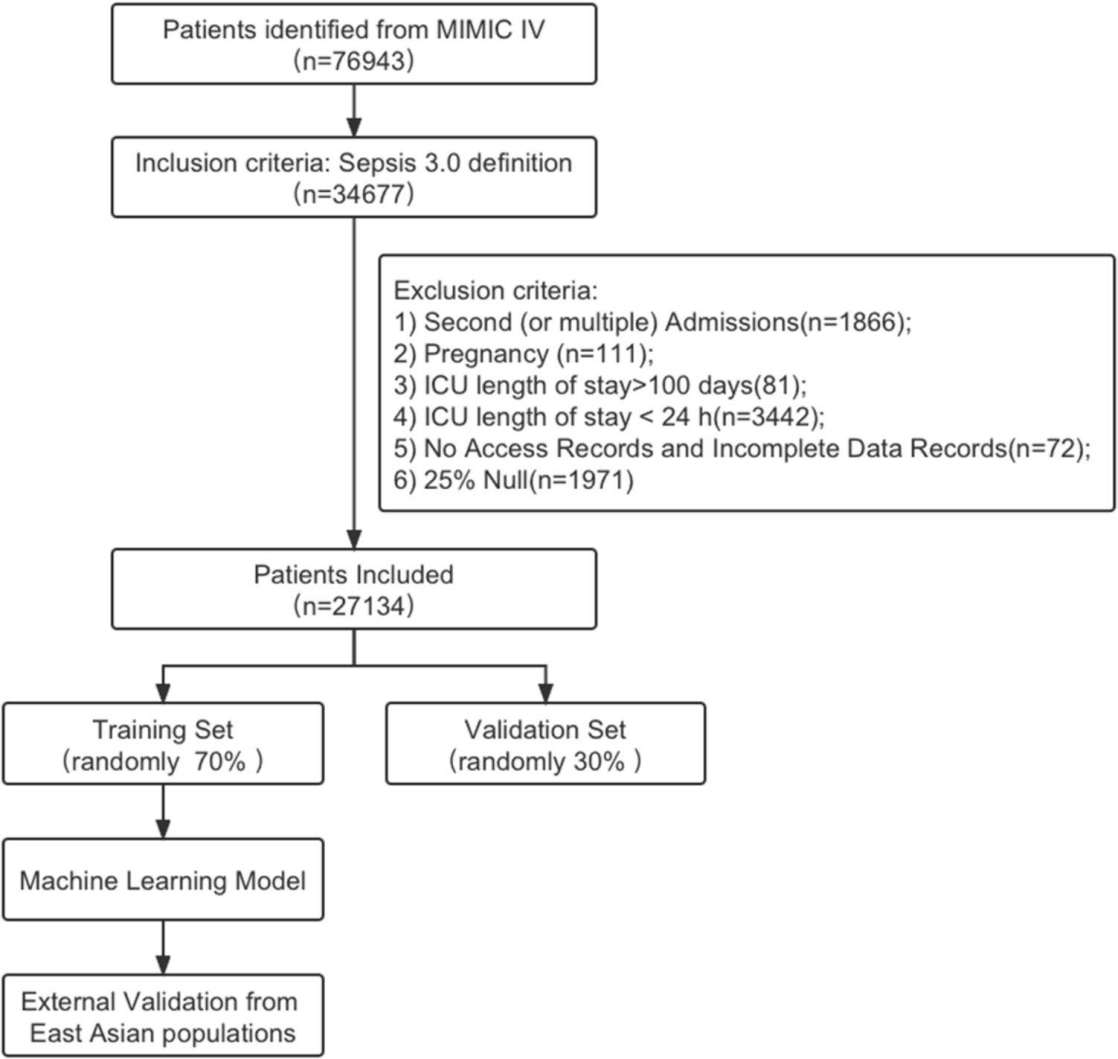
Flowchart of patient selection

Figure 1 depicts the procedure for screening the patient. In the MIMIC-IV database, a total of 76,943 ICU admissions were identified during the initial search. The definition of sepsis was met by a grand total of 34,677 patients. 27,134 eligible patients were included after the exclusion criteria were applied. Table 1 displays the initial clinical and laboratory information of the patients. According to Table 1, a total of 15,900 individuals (58.6%) were identified as male, while the average age of the participants included in the study was 66.6 ± 15.7 years. Of the patients in the MIMIC-IV database, 83.4% (22635) of patients survived. Out of the total 27,134 participants, 4,499 individuals in the study group experienced a mortality rate of 16.6%. According to Table 1, it is evident that individuals who did not survive sepsis tended to be older (65.9 ± 15.6 vs. 70.0 ± 15.1; *P* < 0.01), exhibited reduced systolic blood pressure (113 [105–123] vs. 108 [101 − 120]; *P* < 0.01), and had a higher SOFA score (6 [4–8] vs. 9 [6–12]; *P* < 0.01). The MIMIC-IV database indicates that mortality is linked to elevated levels of white blood cells, creatinine, Lactate, anion gap, as well as increased heart rate and respiratory rate. Additional file 2 presents a comparison of the baseline characteristics and clinical outcomes observed in the local external validation set.

**Table 1:** Baseline characteristics and comparison between survivors and non-survivors in MIMIC database

Demographic characteristics	All *n* = 27,134	Survivors, *n* = 22,635	Non-Survivors, *n* = 4499	*P* value
Demographic information				
Male gender, *n* (%)	15,900 (58.6%)	13,380 (59.1%)	2520 (56%)	< 0.01
Age (yd)	66.6 ± 15.7	65.9 ± 15.6	70.0 ± 15.1	< 0.01
BMI (kg/m^2^)	28 (24–32)	27 (24–32)	26 (24–31)	0.01
Severity of illness				
SOFA [median (IQR)]	6 (4–9)	6 (4–8)	9 (6–13)	< 0.01
SAPS II [median (IQR)]	38 (30–48)	37 (29–45)	49 (39–60)	< 0.01
GCS [median (IQR)]	14 (9–15)	14 (10–15)	10 (4–14)	< 0.01
OASIS [median (IQR)]	35 (28–41)	33 (28–40)	42 (35–48)	< 0.01
Vital signs				
Temperature (℃)	36.9 (36.6–37.2)	36.9 (36.6–37.2)	36.8 (36.5–37.1)	0.01
Systolic blood pressure (mmHg)	112 (104–123)	113 (105–123)	108 (101–120)	< 0.01
Heart rate (bpm)	86 (76–98)	84 (75–96)	90 (78–103)	< 0.01
Respiratory rate (bpm)	19 (16–22)	19 (17–22)	21 (18–24)	0.01
Laboratory findings				
White blood cells (10^9^/L)	13.8 (9.8–18.8)	13.6 (9.8–18.4)	15.0 (10.1–20.9)	0.01
Platelets (10^9^/L)	160 (109–227)	161 (112–226)	154 (87–229)	< 0.01
Creatinine (mg/dl)	1.20 (0.8–1.9)	1.1 (0.8–1.8)	1.6 (1.0–2.7)	< 0.01
Albumin (g/dl)	3.0 (3.0–3.1)	3.0 (3.0–3.1)	2.9 (2.6–3.0)	0.01
Lactate (mmol/L)	2.6 (1.9–2.6)	2.5 (1.9–2.6)	3.6 (2.2–3.8)	< 0.01
Glucose(mg/dl)	132 (113–161)	131 (114–157)	138 (112–178)	0.01
Anion gap	16 (13–19)	16 (13–19)	18 (15–22)	0.01
INR	1.4 (1.2–1.7)	1.4 (1.2–1.6)	1.5 (1.3–2.2)	0.01
Pao2/fio2Ratio	206 (176–216)	208 (184–220)	192 (138–196)	< 0.01
Comorbidity, *n* (%)				
CHD	5078 (18.71%)	4351 (19.2%)	727 (16.2%)	< 0.01
CKD	1753 (6.5%)	1368 (6.0%)	385 (8.6%)	< 0.01
COPD	2800 (10.3%)	2269 (10.0%)	531 (11.8%)	0.01
Liver disease	1653 (6.1%)	1264 (5.6%)	389 (8.6%)	< 0.01
Hypertension	10,135 (37.4%)	8634 (38.1%)	1501 (33.4%)	< 0.01
Diabetes	5096 (18.9%)	4314 (19.1%)	782 (17.4%)	0.01
Treatment status within 24 h, *n* (%)				
Vasopressor (first 24 h)	1919 (7.0%)	1109 (4.9%)	810 (18%)	< 0.01
Mechanical ventilation (first 24 h)	18,187 (33.0%)	14,721 (65%)	3466 (77%)	< 0.01
Renal replacement therapy	1697 (6.2%)	1180 (5.2%)	517 (11.5%)	< 0.01

*SOFA* sequential organ failure assessment, *SAPSII* simplified acute physiology score II, *OASIS* oxford acute severity of illness score, *GCS* glasgow coma scale, *BMI* body mass index, *INR*, international normalized ratio, *CHD* coronary heart disease, *CKD* coronary kidney disease, *COPD* chronic obstructive pulmonary disease

### Evaluation of machine learning algorithm

**Fig. 2:**
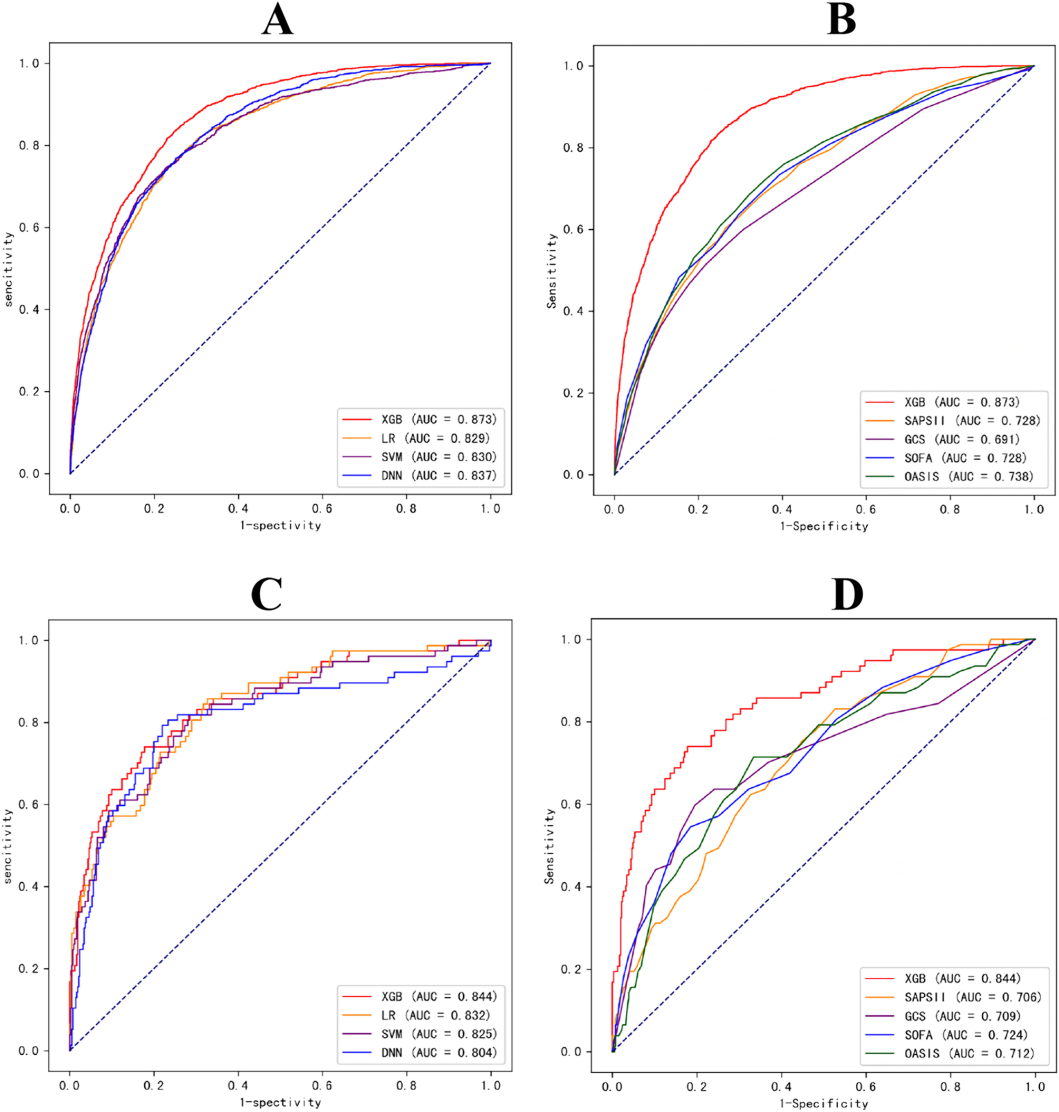
Comparison set of the area under the receiver operating curve. **A** Comparison internal validation set of the area under the receiver operating curve. Performance evaluated by machine learning methods. **B** Comparison internal validation set of the area under the receiver operating curve. XGBoost model compared with clinical scores. **C** Comparison external validation set of the area under the receiver operating curve. Performance evaluated by machine learning methods. **D** Comparison external validation set of the area under the receiver operating curve. XGBoost model compared with clinical scores

To predict mortality in sepsis patients, we employed ML techniques including XGBoost, LR, SVM, and DNN, using all variables as input. The models were trained using the training set obtained from MIMIC-IV. Table 2 displays that the XGBoost model, utilizing all accessible variables, achieved an impressive AUROC of 0.873 [95% CI 0.866–0.880] during the internal validation set and 0.844 (95% CI 0.810–0.878) during the external validation set [15]. Figure 2A displayed that XGBoost demonstrated the highest AUC in the testing dataset (LR, AUC = 0.820; SVM, AUC = 0.831; and DNN, AUC = 0.821). Table 3 displays that the XGBoost model achieved a sensitivity of 0.818, a specificity of 0.768, an accuracy of 0.777, and an F1 score of 0.551. In predicting patient mortality (Fig. 2B), the XGBoost model outperformed the clinical scores SAPS II, SOFA, OASIS and GCS (SAPS II, AUC = 0.728; SOFA, AUC = 0.728; OASIS, AUC = 0.738; GCS, AUC = 0.691). External validation was performed at the First Affiliated Hospital of Wenzhou Medical University, China. Figure 2C and D displayed an AUROC of 0.844 for the XGBoost model during external validation. It is evident that the XGBoost model performed exceptionally well in both the internal and external validation processes.

**Table 2:** Receive operating characteristics curve analysis

	Internal validation set		External validation set	
	AUC	95% CI	AUC	95% CI
XGBoost LR SVM DNN SOFA SAPS II GCS OASIS	0.873 0.829 0.830 0.837 0.728 0.728 0.691 0.738	0.866–0.880 0.820–0.838 0.821–0.839 0.829–0.845 0.717–0.739 0.717–0.739 0.678–0.704 0.727–0.749	0.844 0.832 0.825 0.804 0.724 0.706 0.709 0.712	0.810–0.878 0.796–0.868 0.788–0.862 0.766–0.842 0.678–0.770 0.659–0.753 0.659–0.760 0.666–0.758

**Table 3:** Performance of each model for prediction

	AUC (%)	SE (%)	SP (%)	AC (%)	F1 score	PPV	NPV
XGBoost LR SVM DNN SOFA SAPS II GCS OASIS	0.873 0.829 0.830 0.837 0.728 0.728 0.691 0.738	0.818 0.790 0.745 0.769 0.637 0.604 0.514 0.685	0.768 0.725 0.773 0.749 0.703 0.734 0.783 0.679	0.777 0.736 0.768 0.752 0.731 0.724 0.762 0.702	0.551 0.500 0.518 0.509 0.401 0.409 0.399 0.424	0.818 0.789 0.745 0.768 0.557 0.572 0.466 0.644	0.768 0.725 0.773 0.749 0.764 0.755 0.822 0.713

*SE* sensitivity, *SP* specificity, *AC* accuracy, *PPV* positive predictive value, *NPV* negative predictive value

### Explanation of risk factors

**Fig. 3:**
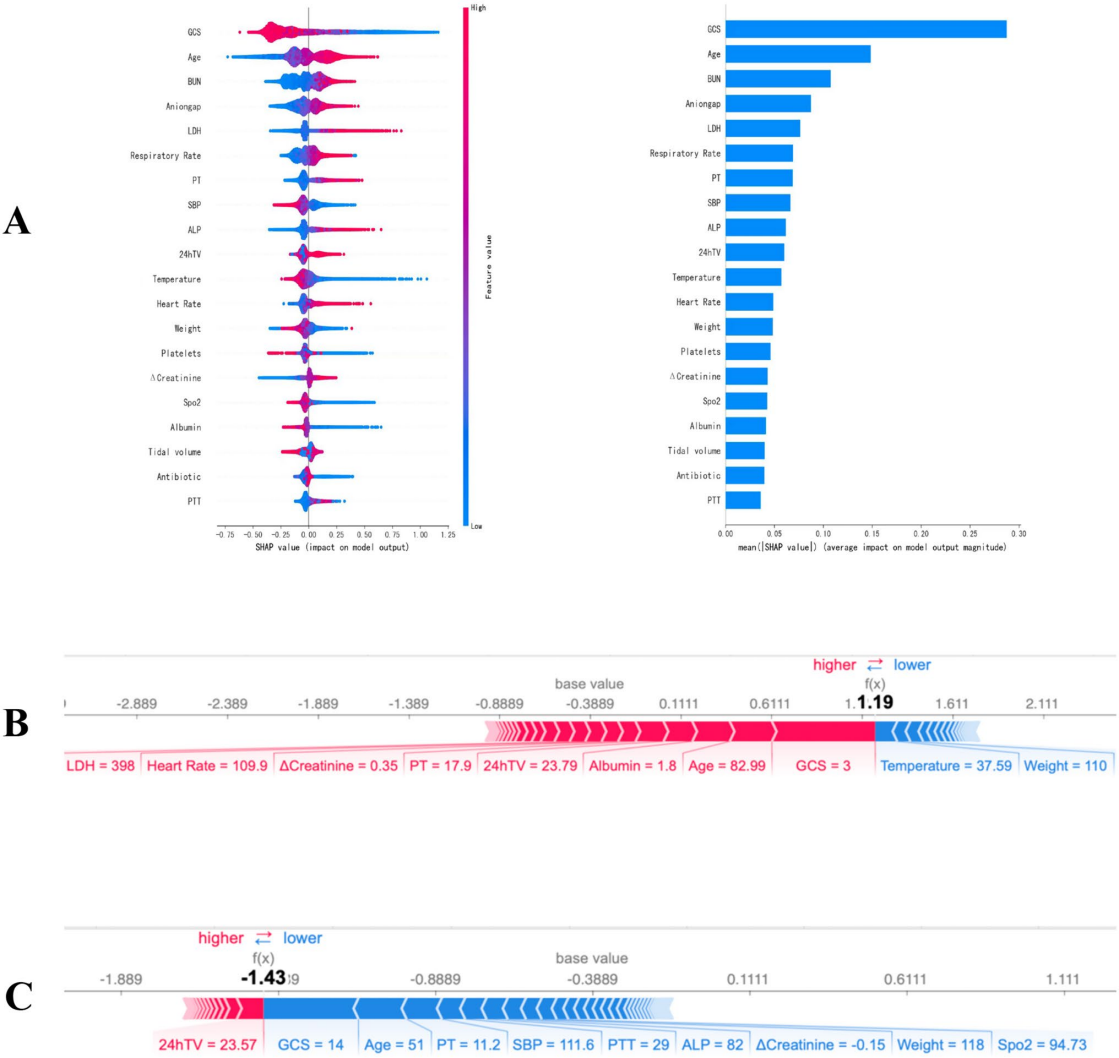
The model’s interpretation. **A** The top 20 variables of the XGBoost model. **B** A high-risk instance. **C** A low-risk instance

Figure 3A displays the evaluated SHAP values of the XGBoost model. In Fig. 3A, the XGBoost model displays the 20 most significant variables, arranged in order of importance. The X-axis of the SHAP value served as a consistent measure for assessing the impact of a feature on the response model. The significance of the prediction model is indicated by the ranking of its features on the Y-axis. In each feature importance row, all patient attributes related to the outcome are visualized using differently colored dots, with red dots representing high values and blue dots representing low values [11]. The SHAP values surpass zero for particular characteristics, indicating an elevated likelihood of fatality. A feature with a higher SHAP value indicates an increased risk of death for the patient. The likelihood of death rises with higher values of the following factors: Age, BUN, Anion gap, LDH, RR, PT, ALP, 24 h ventilation, HR, and average increase in Creatinine. To determine the most influential factors in the prediction model, we illustrated the SHAP summary chart of XGBoost. Figure 3A displays the representation of the individual feature’ contribution to the internal validation set on the right side. Bars of greater length indicate higher significance in relation to the SHAP value. The interpretability of the ML model is demonstrated by Fig. 3B and C, which depict two representative samples. Risk factors and protective factors are represented by the features in red and blue. Figure 3B illustrates a high-risk instance, where the red features contribute to an increase in the risk value of the instance, surpassing the average value calculated by the prediction model [28]. In Fig. 3B, the patient was forecasted to perish due to decreased clinical scores (GCS), advanced age (83 years old), reduced albumin levels (1.8 g/dl), prolonged utilization of mechanical ventilation, along with consistently high PT (17.9 s) and creatinine (average increase). The instance has a low level of risk, as the blue features decrease the risk value of the instance below the average value. In Fig. 3C, the patient was anticipated to be living due to elevated clinical scores (GCS) and being younger (51 years old).

## Discussion

This study developed and validated a ML model to predict mortality in sepsis patients. The model’s performance was assessed on two study groups from the MIMIC-IV database and a Chinese teaching hospital, demonstrating robustness across different datasets. Compared to previous studies, our approach utilized the more comprehensive MIMIC-IV dataset, covering a wider time span and a larger number of hospital admissions.

We employed multiple machines learning techniques and found that the XGBoost model outperformed LR, SVM, and DNN, as well as conventional clinical scores. The model achieved an impressive AUROC of 0.873 and showed high specificity, accuracy, and sensitivity in both internal and external validation. Our ML model demonstrated exceptional reliability and prospective generalization ability in both the internal and external validation sets, surpassing the prediction performance of conventional methods significantly. Additionally, medical professionals can utilize the SHAP dependency graph to promptly make informed choices that facilitate suitable medical intervention and enhance the efficient allocation of healthcare resources [11]. If a patient is at a high risk of mortality, it is imperative to administer intensive medical intervention [28]. Expanding these methods to patients in other medical or surgical departments could enhance their utility in predicting complications and mortality, thus aiding in ICU admission decisions.

With the flexibility to incorporate diverse clinical criteria and data attributes [29], ML can construct predictive models for various medical scenarios, including the prognosis of cardiac arrest in septic patients [30, 31], forecasting multiple organ function in elderly individuals [32, 33], predicting the risk of premature mortality in critically ill patients [29], and anticipating readmission outcomes for those in intensive care [34]. Presently, numerous associated prototypes employ artificial intelligence (AI) to forecast sepsis [9, 35], thereby augmenting doctors’ capacity for medical decision-making concerning patients afflicted with sepsis [15]. Hu et al. aimed to develop and validate an interpretable machine-learning model using data from 8817 sepsis patients in the MIMIC-IV database [36]. Leveraging information from modern electronic medical records, ML techniques can be utilized to predict the early occurrence of sepsis [9], as demonstrated by Nemati et al.

Despite the numerous studies and models available in the literature regarding mortality prediction in sepsis patients, local validation of such algorithms based on the MIMIC database was lacking. Our approach addresses limitations of existing severity scores, offering an early and accurate tool for mortality prediction in sepsis patients. This model’s accessibility to readily available admission variables can aid clinicians in making timely interventions [4, 37]. To the best of our understanding, this study is one of the earliest attempts to predict premature death in sepsis patients using a comprehensive public database and a locally validated database [15, 38, 39]. We are able to modify these ML models to incorporate real time change and provide clinical decision support to the clinical team.

Our study also had several limitations. Initially, considering the aspect of model development, we have utilized certain dynamic time series data. To improve the accuracy of sepsis outcome predictions, we plan to construct a dynamic model in future and include additional features [40]. Second, Furthermore, due to the retrospective nature, single-center data could induce selection bias, warranting multi-center investigations for broader validation [41]. To enhance the model’s universality, more diverse data sources are needed, despite Chinese hospital validation. In future, once verified through a local database and with ongoing parameter adjustments, the model will be able to provide enhanced support to clinicians when making clinical decisions.

## Conclusion

In conclusion, machine learning holds substantial promise in sepsis. Our developed model for early sepsis mortality prediction using machine learning demonstrated impressive performance, as validated externally in a Chinese teaching hospital, yielding consistent results.

## Data Availability

The raw data supporting the conclusions of this article will be made available by the authors, without undue reservation. The extracted data and programming code for model development in this study are available from the corresponding author on reasonable request. Data of the MIMIC- IV are available on website [42]. It is an open database.

## Abbreviations

SOFA: Sequential organ failure assessment
SAPS II: Simplified acute physiology score II
GCS: Glasgow coma scale
APACHE II: Acute physiology and chronic health assessment scoring system II
OASIS: Oxford acute severity of illness score
qSOFA: Quick sequential (sepsis-related) organ failure assessment
BMI: Body mass index
INR: International normalized ratio
CHD: Coronary heart disease
CKD: Coronary kidney disease
COPD: Chronic obstructive pulmonary disease
SE: Sensitivity
SP: Specificity
AC: Accuracy
AUROC: Area under the receiver operating characteristic curve
ALP: Alkaline phosphatase
PT: Prothrombin time
LDH: Lactate dehydrogenase

## Declarations

### Provenance and peer review

Not commissioned, externally peer reviewed.
